# Development and validation of super learner models to predict small and large for gestational age in the second generation

**DOI:** 10.1038/s41598-025-18466-0

**Published:** 2025-09-26

**Authors:** Mary M. Brown, Stefan Kuhle, Bruce Smith, Victoria M. Allen, Jennifer Payne, Christy G. Woolcott

**Affiliations:** 1 School of Integrated Health, University of New Brunswick, Saint John, NB Canada; 2 Medical Statistics and Informatics, Institute of Clinical Epidemiology, Public Health, Health Economics, Medical University of Innsbruck, Innsbruck, Austria; 3 Perinatal Epidemiology Research Unit, Depts of Obstetrics & Gynaecology and Pediatrics, Dalhousie University, Halifax, NS Canada; 4 Dept of Mathematics and Statistics, Dalhousie University, Halifax, NS Canada; 5 Dept of Obstetrics & Gynaecology, Dalhousie University, Halifax, NS Canada; 6 Dept of Diagnostic Radiology, Dalhousie University, Halifax, NS Canada

**Keywords:** Birthweight, Small for gestational age, Large for gestational age, Intergenerational factors, Pregnancy, Prediction, Paediatric research, Epidemiology

## Abstract

Prediction of small (SGA) and large for gestational age (LGA) using routinely collected antenatal data remains suboptimal, particularly among nulliparous women. In this study, models for SGA (< 10^th^ percentile) and LGA (> 90^th^ percentile) were developed by combining grandmaternal pregnancy-related information and maternal birth characteristics (“G0 predictors”) with maternal clinical factors available at 26 weeks’ gestation (“G1 predictors”). The study used a cohort of first-born, singleton births to nulliparous women in Nova Scotia, Canada (1981–2011), and their mothers, from the Nova Scotia Atlee Perinatal Database. Models using G0 predictors, G1 predictors, and their combination were developed with Super Learner, an ensemble machine learning algorithm, and internally validated using nested cross-validation. Discrimination was assessed via the area under the receiver operating characteristic curve (AUC-ROC) and the precision-recall curve (AUC-PR); calibration was also evaluated. Among 9,097 grandmother-mother-infant triads, 902 (9.9%) infants were SGA and 891 (9.8%) were LGA. Including G0 predictors improved discrimination compared to G1-only models (AUC-ROC 0.69 vs. 0.66 for SGA and 0.71 vs. 0.66 for LGA; AUC-PR: 0.21 vs. 0.18 for SGA and 0.22 vs. 0.18 for LGA). Models fitted using both sets of predictors were well calibrated. While incorporating intergenerational information modestly improved prediction, overall predictive performance remains poor.

**Supplementary Information:**

The online version contains supplementary material available at 10.1038/s41598-025-18466-0.

## Introduction

Deviations from normal fetal growth are associated with adverse perinatal and long-term health outcomes. Infants born small for gestational age (SGA) have increased risks of perinatal morbidity and mortality, neurodevelopmental deficits, and cardiovascular disease in later in life^1–3^. Those born large for gestational age (LGA) are faced with increased risk of birth injury, asphyxia, polycythemia, and hypoglycemia, and are more likely to develop obesity, diabetes, and cardiovascular disease in adulthood^1,4^. Accurate identification of pregnancies at risk for fetal growth abnormalities may enhance preconception counselling, antenatal assessment, and intrapartum care.

Several prediction models for SGA and LGA have been developed using routinely collected antenatal data, including maternal sociodemographic characteristics, pregnancy risk factors, obstetric history, and clinical characteristics; however, predictive performance remains poor, particularly among nulliparous women. For example, one validation study of six prediction models for SGA and LGA using a cohort of 1,311 nulliparous women reported discriminative performance estimates, measured as the area under the receiver operating characteristic curve (AUC-ROC), of 0.50–0.66 for SGA and 0.58–0.67 for LGA^5^. Similarly, in a cohort of 14,923 nulliparous Nova Scotian women, prediction models resulted in AUC-ROC estimates of 0.63 for SGA and 0.70 for LGA^6^. Efforts to improve prediction models for early detection of SGA and LGA include adding ultrasound measurements, biochemical markers, and results of biophysical tests, but only modest improvements have been reported^7–14^ and measurement of some predictors may be costly, time-consuming, and inconvenient for pregnant women^15^.

Fetal growth is shaped by a complex combination of maternal, fetal, and environmental factors. Recent evidence has suggested that grandparental characteristics may also play a role, with studies reporting small to moderate associations between grandparental risk factors and child birthweight^16^, including grandparental birthweight^17,18^, body mass index (BMI)^19,20^, smoking in pregnancy^21,22^, socioeconomic status^23^, and diabetes^24,25^, with most research focusing on the maternal line. Despite the well-established relationship between maternal and offspring size-at-birth^26^, maternal birth characteristics and grandmaternal pregnancy-related information have not yet been incorporated into prediction models for SGA or LGA.

The application of machine learning algorithms to predict clinical outcomes is growing^27,28^, but remains relatively limited in the context of SGA and LGA^6,15,29^. Unlike traditional regression-based approaches to prediction, machine learning methods learn from existing data without explicit model specification, make fewer distributional assumptions, and can capture non-linear relationships and complex interactions among predictors. Given that grandmaternal factors may modify maternal-offspring associations and that these relationships may be non-linear, machine learning approaches may offer improved predictive performance in this setting.

Due to the difficulty in determining a priori which machine learning algorithm will perform best in a given dataset, predictive performance can be optimized by combining predictions from multiple algorithms using ensemble learning methods. Therefore, the objective of this study was to develop and validate prediction models for SGA and LGA by integrating grandmaternal pregnancy-related information and maternal birth characteristics (“G0 predictors”) with maternal clinical factors available at 26 weeks’ gestation (“G1 predictors”) using the ensemble machine learning algorithm Super Learner in a large sample of nulliparous women in Nova Scotia, Canada.

## Methods

### Study population and design

Data were derived from the 3G Multigenerational Cohort^30^, which includes women whose births and subsequent pregnancies were recorded in the Nova Scotia Atlee Perinatal Database (NSAPD). The NSAPD is a population-based database that contains extensive information on demographics, medical conditions, reproductive history, delivery events, and neonatal outcomes for each birth to mothers residing in Halifax County, Nova Scotia, Canada, since 1981, and to mothers residing anywhere in the province after 1988. Information is collected from standard forms completed prenatally and during the hospital stay associated with the delivery. Provincial health card numbers are assigned to all Nova Scotia residents at birth and remain with them throughout their lifetime, even if they leave and return to the province. Health card numbers of women and their offspring are recorded in the NSAPD thereby facilitating creation of the 3G Multigenerational Cohort by linking women’s birth information with information on their own pregnancies and deliveries. The Reproductive Care Program has assigned many health card numbers retroactively for those born before the introduction of health card numbers in 1993 based on name, date of birth, civic address, medical records, and later deliveries in the database.

As of April 30th, 2021, the 3G cohort consisted of 19,583 grandmothers (born 1939–1987), 22,307 mothers (born 1981–2006), and 38,922 infants (born 1996–2021). The present study restricted the cohort to singleton pregnancies and the first-born offspring in both the maternal and grandmaternal generations. In addition, only second-generation infants with complete information on gestational age and birthweight, gestational age $\ge$ 26 weeks, and a plausible value of birthweight z-score (< 5 in absolute value) were included in the analysis.

### Outcomes

The two primary outcomes of interest were infant birthweight for gestational age and sex: SGA (< 10^th^ percentile) and LGA (> 90^th^ percentile) relative to a Canadian reference population^31^. Secondary outcomes included more stringent definitions (< 3^rd^ percentile (SGA3) and > 97^th^ percentile (LGA97) for gestational age). Birthweight was recorded in grams on the birth record. Gestational age was available in days and was estimated using information from a dating ultrasound, the last menstrual period, and where applicable, embryo transfer; details of the algorithm can be found elsewhere^32^.

### Predictors

Two sets of predictors were considered (Supplementary Table S1). “G1 predictors” included the mother’s demographic, pre-pregnancy, and pregnancy information that was available at 26 weeks’ gestation. “G0 predictors” included grandmaternal demographic, pregnancy, and delivery characteristics at the time of the mother’s birth and the mother’s birth characteristics and neonatal outcomes.

Area-level income quintile was used as a measure of socioeconomic status and was derived from linkage of the woman’s residence postal code to national census information^33^. Blood pressure was measured at each prenatal visit to screen for pre-existing hypertension (< 20 weeks’ gestation) and hypertensive disorders of pregnancy (onset $\ge$ 20 weeks’ gestation, pre-existing hypertension with superimposed proteinuria, or eclampsia) based on the Society of Obstetricians and Gynaecologists of Canada Guidelines^34^. All women in Nova Scotia were eligible to undergo screening for gestational diabetes according to guidelines set by Diabetes Canada^35^. Any smoking reported during pregnancy (first prenatal visit, 20 weeks, or birth admission), or any alcohol use disorder reported at any point in the pregnancy, were used as proxy measures for smoking and alcohol use disorders at 26 weeks. Pre-pregnancy BMI was calculated by dividing pre-pregnancy weight (kg) by the square of height (m), which are recorded at the first prenatal visit. Gestational weight gain (kg) at 26- and 40-weeks’ gestation (denoted by $X$ below) was estimated by

$$2+(X-13)\left(\frac{\text{Delivery weight (kg)}-\text{Pre-pregnancy weight (kg)}-2}{\text{Gestational age at birth (weeks)}-13}\right)$$

assuming a 2 kg weight gain in the first trimester (13 weeks) and a steady increase in weight thereafter^36^.

### Statistical analysis

Descriptive statistics including means and standard deviation and percentages were used to describe the study sample overall and by SGA and LGA status. Absolute standardized mean differences (SMD)^37^ between groups defined by SGA and LGA status were computed for each predictor. Continuous predictors were standardized (rescaled to have a mean of 0 and a standard deviation of 1) prior to model building.

Prediction models were developed using G1 predictors only, G0 predictors only, and their combination. All models were developed using the Super Learner algorithm^38^, an ensemble method that combines predictions from multiple models into a single model that has been optimized for predictive accuracy. Optimization occurs by assigning weights based on each algorithms performance with respect to a user-specified loss function; in this study, the Brier score (equivalent to the mean squared error for predictions) was used. The resulting Super Learner model has been shown to perform as well as, or better than, the best algorithm in the ensemble in large samples^39^.

The library of candidate learners included a range of statistical and machine learning approaches: logistic regression (with and without interaction terms); generalized additive models (GAMs), an extension of generalized linear models that allow for flexible, non-linear relationships between predictors and the dependent variable; elastic net^40^, a regularized regression technique that performs well with highly correlated predictors; random forest^41^, an ensemble of decision trees; tree-based extreme gradient boosting (XGBoost)^42^, a more refined tree-based method that improves accuracy by correcting errors made by the previous trees built in the model (boosting); and kernel-based support vector machine (SVM)^43^, which classify observations by identifying optimal decision boundaries in the data. This set of learners was selected to provide a diverse set of modeling approaches while remaining computationally feasible.

For each imputed dataset, nested stratified cross-validation (tenfold inner and fivefold outer cross-validation) was used to develop and validate the prediction models. For learners requiring hyperparameter tuning, Super Learner (fitted using fivefold cross-validation) was used to create an optimally weighted combination of the learner fitted with different hyperparameter configurations (Supplementary Table S2), with weights optimized based on the Brier score. Predictions from the base learners (including the tuned learners) were then passed to the main Super Learner, which was fitted and evaluated using the nested cross-validation framework. Models were evaluated based on discrimination and calibration. Discrimination was assessed using the AUC-ROC and the area under the precision-recall curve (AUC-PR), which is a plot of precision (i.e., positive predictive value) vs. recall (i.e., sensitivity). A lack of discrimination is indicated by an AUC-PR value equal to the prevalence of the outcome. Predictive variable importance rankings in the two estimation algorithms with the largest mean weight in the Super Learner ensemble were measured by the increase in Brier score after permuting the values of each predictor and averaging across imputed datasets.

Calibration, the agreement between predicted and observed risk, was visually assessed using calibration curves fitted using thin plate splines and decile groups. Data for the calibration curves were created by stacking the Super Learner predictions from each imputed dataset when each observation served in the validation fold. Within each imputed dataset, deciles calculated using the stacked Super Learner predictions were used to group observations, within which the proportion of observations with the outcome was estimated. Pooled estimates were used to derive calibration point estimates with 95% confidence intervals (CI).

All analyses were performed in R (v4.2.1)^44^ using functions primarily from the *mice,*^45^ *psfmi,*^46^* PRROC,*^47,48^ *SuperLearner*^49^, and *ck37r*^50^ packages and modified R source code developed by Chris Kennedy^51^. This study followed the Transparent Reporting of a Multivariable Prediction Model for Individual Prognosis or Diagnosis plus Artificial Intelligence (TRIPOD + AI) reporting guidelines^52^.

### Missing data

Implausible values of maternal birthweight z-score ($\ge$ 5 in absolute value) and grandmaternal and maternal pre-pregnancy weight (BMI < 13 kg/m^2^, or < 35 kg if height was missing) were set to missing. The proportion of missing values was below 32% for all predictors, except for grandmaternal pre-pregnancy height and consequently, BMI, where approximately 90% of values were missing due to height information not being routinely collected before 2003. Multiple imputation using chained equations was used to account for missingness in predictors^45^. Imputation models included G0 predictors, G1 predictors, and outcome variables. Ten imputed datasets (25 iterations) were generated where missing values for each variable with missingness were imputed using random forest (RF)^41,53^. Continuous derived variables, such as BMI and gestational weight gain, were passively imputed^54^. Analyses were performed on each imputed dataset and results were pooled using Rubin’s rules^55^. Traceplots suggested no issues with convergence of the multiple imputation procedure, and a comparison of imputed and observed values showed that the imputed values were all within reasonable ranges and did not differ substantially from the observed values.

## Results

### Study population

**Fig. 1:**
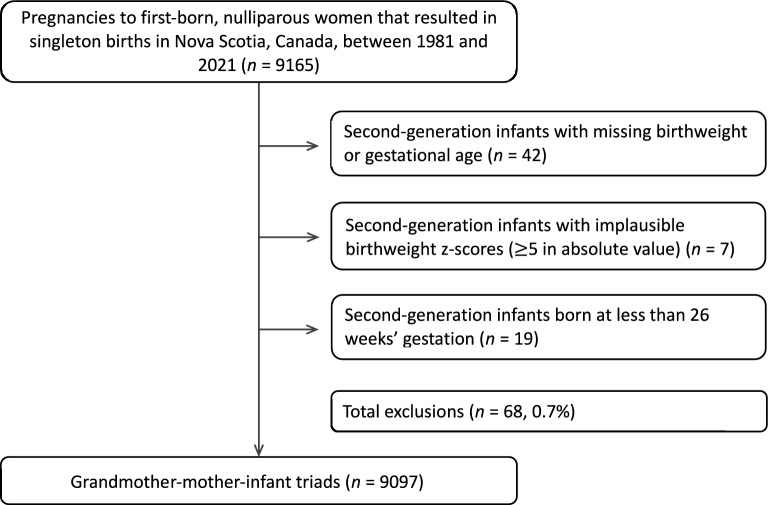
Flow chart describing study cohort creation.

Between 1981 and 2021, a total of 9,165 pregnancies to first-born, nulliparous women resulted in singleton births. After removing second-generation infants with missing birthweight or gestational age (n = 42) or implausible birthweight z-scores (n = 7), and those born at less than 26 weeks’ gestation (n = 19), the final analytical sample included 9,097 grandmother-mother-infant triads (Fig. 1). During this period, 902 (9.9%) and 891 (9.8%) infants were born SGA and LGA, respectively. Characteristics of the sample are shown in Table 1. Small differences (absolute SMDs less than 0.2) in grandmaternal characteristics existed by outcome status. Mothers of infants born SGA had lower birthweight z-scores and, as adults, had lower pre-pregnancy BMIs and gained less weight in pregnancy than mothers of infants born non-SGA. Mothers of infants born LGA had higher birthweight z-scores and, as adults, had higher pre-pregnancy BMIs and gained more weight in pregnancy than mothers of infants born non-LGA.

**Table 1:** Sample characteristics overall and by small for gestational age and large for gestational age.

	Complete, n	**Overall**^**a**^ **(n = 9097)**	**SGA**^**a**^** (< 10**^**th**^** percentile for gestational age)**			**LGA**^**a**^** (> 90**^**th**^** percentile for gestational age)**		SMD
			No (n = 8195)	Yes (n = 902)	SMD	No (n = 8206)	Yes (n = 891)	
**Grandmaternal characteristics**								
Maternal age [years]	9097	23.6 (4.6)	23.6 (4.5)	23.5 (4.7)	0.02	23.5 (4.6)	23.9 (4.6)	0.07
Married or common-law	8774	60.4	60.6	59.0	0.03	60.2	62.6	0.05
Area-level income quintile	6388				0.05			0.06
Low (Q1)		25.8	25.8	25.3		26.0	23.6	
Middle (Q2-Q4)		63.8	63.6	65.4		63.6	65.5	
High (Q5)		10.4	10.6	9.3		10.4	10.8	
Rural residence	6417	35.4	34.8	41.1	0.13	35.5	34.6	0.02
Pre-pregnancy body mass index [kg/m^2^]	626	23.2 (4.8)	23.2 (4.8)	22.9 (4.3)	0.08	23.1 (4.8)	23.8 (4.6)	0.14
Pre-existing hypertension	9097	0.7	0.7	0.6	0.02	0.7	0.8	0.01
Weight gain in pregnancy at 40 weeks [kg]	7167	15.4 (6.3)	15.5 (6.3)	14.5 (6.9)	0.14	15.3 (6.4)	16.4 (5.8)	0.18
Any smoking in pregnancy	8027	40.9	40.6	43.8	0.07	41.0	40.1	0.02
Any alcohol use in pregnancy	7830	14.5	14.4	15.2	0.02	14.6	13.3	0.04
Gestational diabetes	9097	1.8	1.8	1.9	0.01	1.8	1.9	0.01
Hypertensive disorders of pregnancy	9097	14.4	14.3	15.2	0.03	14.3	15.3	0.03
Caesarean section	9091	18.4	18.7	15.4	0.09	17.8	23.6	0.14
**Maternal birth characteristics**								
Born LGA (> 90^th^ percentile for gestational age)	8877	7.2	7.7	3.1	0.21	6.2	16.7	0.34
Born SGA (< 10^th^ percentile for gestational age)	8877	13.3	12.3	22.2	0.26	14.0	7.0	0.23
Birthweight z-score [SD units]	8877	−0.19 (1.0)	−0.15 (1.0)	−0.59 (1.0)	0.44	−0.24 (1.0)	0.27 (1.1)	0.50
**Maternal characteristics in adulthood**								
Maternal age [years]	9097	23.9 (4.5)	24.0 (4.5)	23.5 (4.3)	0.11	23.9 (4.5)	24.1 (4.5)	0.05
Married or common-law	8127	45.5	46.1	40.1	0.12	45.1	49.5	0.09
Area-level income quintile	8785				0.07			0.01
Low (Q1)		22.4	22.4	22.3		22.4	22.1	
Middle (Q2-Q4)		66.6	66.4	68.5		66.6	66.6	
High (Q5)		11.0	11.2	9.2		11.0	11.3	
Rural residence	8806	33.6	32.8	40.8	0.17	33.9	30.9	0.06
Pre-pregnancy body mass index [kg/m^2^]	7645	26.0 (6.7)	26.1 (6.7)	24.6 (6.4)	0.23	25.8 (6.6)	27.8 (7.2)	0.29
Pre-existing hypertension	9097	0.9	0.9	0.9	0.00	0.9	1.3	0.05
Pre-existing diabetes	9097	0.9	0.9	0.6	0.05	0.7	2.9	0.17
Weight gain in pregnancy at 26 weeks [kg]	6892	8.9 (4.2)	9.1 (4.2)	7.8 (3.7)	0.31	8.8 (4.1)	10.2 (4.5)	0.32
Smoking in pregnancy at 26 weeks	9029	23.2	21.9	34.7	0.29	24.2	13.2	0.29
Alcohol use in pregnancy at 26 weeks	9097	1.1	1.1	1.2	0.02	1.1	0.8	0.03
Gestational diabetes	9097	4.2	4.2	4.0	0.01	4.0	5.4	0.06
Hypertensive disorders of pregnancy	9097	10.5	10.2	13.2	0.09	10.4	11.9	0.05
**Infant characteristics**								
Male sex	9097	51.9	51.2	58.1	0.14	51.9	51.7	0.00

*LGA* large for gestational age; *SD* standard deviation; *SGA* small for gestational age; *SMD* absolute standardized mean difference.

^a^ Continuous variables summarized using means (SDs) and categorical variables summarized using percentages.

### Discrimination

Cross-validated estimates of the AUC-ROC and AUC-PR for the Super Learner algorithm are shown in Table 2. For both SGA and LGA, AUC estimates from models fitted using the combined set of predictors were poor, with an AUC-ROC of 0.69 for SGA and 0.71 for LGA, and an AUC-PR of 0.21 for SGA and 0.22 for LGA. For reference, an AUC-ROC of 0.5 and an AUC-PR of approximately 0.1 (average prevalence of SGA and LGA in training samples) suggest no discrimination. These estimates were only marginally higher than those obtained from models fitted using either predictor set alone (AUC-ROC 0.63–0.66 for SGA and 0.64–0.66 for LGA; AUC-PR 0.15–0.18 for SGA and 0.17–0.18 for LGA). ROC and precision-recall curves can be found in Supplementary Figure S1 and Supplementary Figure S2. AUC estimates for the Super Learner algorithm and the individual learners are shown in Supplementary Table S3.

**Table 2:** Cross-validated discriminative performance of the Super Learner algorithm for predicting small for gestational age and large for gestational age fitted using grandmaternal pregnancy-related information and maternal birth characteristics (G0 predictors), maternal clinical factors at 26 weeks’ gestation (G1 predictors), and their combination (G0 + G1 predictors).

	**AUC-ROC (95% CI)**	**AUC-PR (95% CI)**	**Mean Brier Score**^**a**^
**Small for gestational age**			
G0 predictors	0.63 (0.61, 0.65)	0.15 (0.14, 0.17)	0.0877
G1 predictors	0.66 (0.64, 0.68)	0.18 (0.16, 0.21)	0.0864
G0 + G1 predictors	0.69 (0.67, 0.71)	0.21 (0.19, 0.24)	0.0850
**Large for gestational age**			
G0 predictors	0.64 (0.62, 0.67)	0.17 (0.15, 0.18)	0.0862
G1 predictors	0.66 (0.64, 0.68)	0.18 (0.16, 0.20)	0.0857
G0 + G1 predictors	0.71 (0.69, 0.73)	0.22 (0.19, 0.24)	0.0837

*AUC-PR* area under the precision-recall curve; *AUC-ROC* area under the receiver operating characteristic curve; *CI* confidence interval.

^a^ Mean Brier score across validation folds and imputed data sets.

### Calibration

**Fig. 2:**
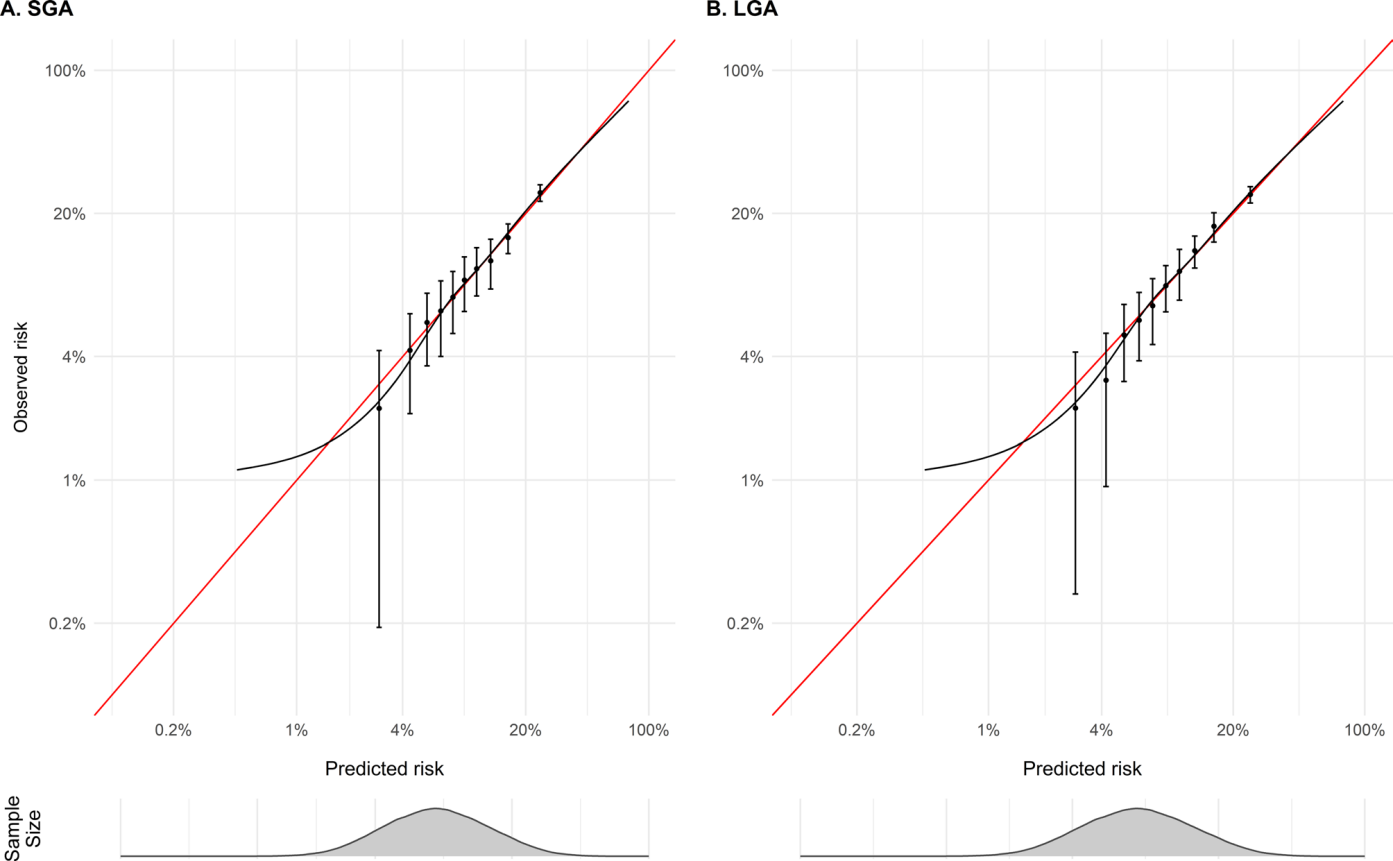
Calibration plots showing the comparison of predicted risk from Super Learner algorithms fitted using the combined set of predictors (G0 + G1 predictors) and deciles of observed risk plotted on the logarithmic scale for **A**) small for gestational age (SGA) and **B**) large for gestational age (LGA). The red line indicates perfect calibration.

The mean predicted risk of SGA and LGA from the Super Learner ensemble (approximately 10% for both) matched the overall risk in the sample. Calibration plots (Fig. 2) indicated good agreement between the predicted risk of SGA and LGA from the Super Learner and the smoothed actual risk estimated using thin plate splines. The Super Learner ensemble slightly overestimated the risk of SGA and LGA when the actual risk was small (< 5%) (Supplementary Table S4) but predicted risk estimates were within the 95% CIs for the actual risk in all decile groups.

### Super learner weights and variable importance

To assess the contribution of the individual learners to the final Super Learner predictions, coefficients (i.e., weights) of the Super Learner model were calculated and averaged across validation folds and imputed datasets (Supplementary Table S5). Most highly weighted in SGA prediction were XGBoost (mean weight 0.41), elastic net (mean weight 0.18), and GAM (mean weight 0.18). Most highly weighted in LGA prediction were GAM (mean weight 0.43), RF (mean weight 0.25), and XGBoost (mean weight 0.21). Similar key predictors were identified across outcomes and estimation methods (Supplementary Table S6); all were related to the maternal generation: birthweight z-score, gestational weight gain, and pre-pregnancy BMI.

### Sensitivity analysis

The prevalence of SGA3 and LGA97 was 2.6% and 3.3%, respectively, which represented the value of no discrimination for AUC-PR. Super Learner predictions were best with the combined set of G0 and G1 predictors (Supplementary Table S7). Discriminative performance for SGA3 was similar to that for SGA defined by the 10^th^ percentile, but slightly better for LGA97 than for LGA defined by the 90^th^ percentile. Key predictors for SGA3 and LGA97 (Supplementary Table S8) were similar to those identified in the primary analyses.

## Discussion

The current study used a large sample of prospectively collected data on three generations to assess the addition of grandmaternal pregnancy-related information and maternal birth characteristics to Super Learner models for SGA and LGA based on standard maternal factors in nulliparous women. Predictive performance measured using the AUC-ROC and AUC-PR increased with the inclusion of grandmaternal pregnancy-related factors and maternal birth characteristics to models fitted using only maternal characteristics, but discriminative performance remained poor. Predictors most influential on model performance included maternal clinical factors such as weight gain in pregnancy at 26 weeks and pre-pregnancy BMI, but also the mother’s birthweight z-score, a factor rarely considered in the prediction of SGA and LGA.

This study focused on using easily obtainable antenatal information to predict SGA and LGA. Other prediction models based on maternal characteristics alone have reported similar AUC-ROC estimates between 0.59 and 0.75 for SGA and LGA^6,7,9–11,13,14,56–59^, but few have been restricted to nulliparous women^5,6,10,11^ in whom prediction is poorer^5,6^. The AUC-PR estimates from the present study could not be compared to other studies in which this measure of discrimination was not reported.

Prediction of SGA and LGA was only slightly improved by the addition of grandmaternal factors and maternal birth characteristics. As several studies have shown an association between a mother’s own birthweight and the intrauterine growth of her offspring^18,26,60^, the observed increase in performance may be attributed to the addition of maternal birthweight z-score. For instance, a meta-analysis of three studies reported a 2.6 times increase in the odds of having a SGA birth in women who themselves were born SGA compared to women who were born non-SGA^61^. Moreover, a study using the Swedish Birth Register indicated that women who were born LGA had twice the odds of having an LGA infant in their own pregnancy^62^. In the current study, maternal SGA and LGA status was associated with a two and nearly two and a half times increased risk of having a (first-born) SGA and LGA birth, respectively, compared to mothers born non-SGA and non-LGA.

Only two studies have considered maternal birthweight in prediction models for SGA or LGA but model discrimination was poor (AUC-ROC 0.63 for SGA^10^, and 0.59 for LGA^11^). In the current study, maternal birthweight z-score was consistently identified as an important predictor of both outcomes. In an exploratory analysis, the Super Learner algorithm fitted using maternal predictors and maternal birthweight z-score (i.e., ignoring all other G0 predictors) performed as well as models that included all grandmaternal predictors. Thus, maternal birthweight z-score likely contributed to the increase in predictive performance seen with the addition of all G0 predictors as a set.

The primary outcomes in this study were defined using the 10^th^ and 90^th^ percentiles for birthweight-for-gestational age and sex, consistent with definitions used in other studies of fetal growth. However, there is growing concern that these thresholds do not adequately capture true fetal growth restriction or excessive fetal growth^63,64^. Although lower thresholds may better identify infants at highest risk of adverse outcomes, the aim of this study was not to detect pathologic growth, but to evaluate whether intergenerational pregnancy-related information provides additional predictive value beyond standard clinical factors. In sensitivity analyses, we found that model performance and variable importance were similar when using the 3^rd^ and 97^th^ percentiles, suggesting minimal impact of the specific thresholds used. Despite limitations in the definition of SGA and LGA, these classifications remain clinically relevant: they are associated with increased risk of adverse perinatal morbidity, and are easily operationalized, making them practical outcomes for prediction tools in clinical settings.

The use of data-adaptive algorithms to predict health outcomes is becoming more popular^27,28^. Many data-adaptive algorithms have the theoretical advantage over parametric models like logistic regression in that they require no distributional assumptions, no explicit model specification, and can capture non-linear relationships between the predictors and the outcome. However, in the present study and another study conducted in the same population^6^, AUC estimates from the Super Learner were approximately the same or only minimally higher than those derived from logistic regression models. With the inclusion of continuous predictors that could have non-linear associations with the outcomes, it was expected that the Super Learner algorithm would perform better than logistic regression. Prediction models developed using GAM performed nearly as well as the Super Learner algorithm, suggesting non-linear associations were likely accommodated by splines and relevant interactions among predictors did not exist. The Super Learner algorithm should still be considered in other datasets that may be more complex, contain a larger number of predictors, or when interactions among predictors are likely.

A main strength of this study is the use of a large sample with prospectively collected data on a diverse set of variables from three generations of Nova Scotians. A second strength is the flexible modeling approach used to predict SGA and LGA, which reduced the risk of bias due to model misspecification. This study also has several limitations worth discussing. First, approximately 90% missingness in grandmaternal pre-pregnancy height (required to calculate BMI) occurred because height has only been routinely collected in the NSAPD since 2003. However, analyses of multiply imputed BMI values were expected to be minimally biased since height was likely to be missing at random, and the imputation procedure included variables that were correlated with height. This study was also limited by the availability of predictors in the NSAPD, so other early-pregnancy factors such as paternal characteristics could not be included.

In conclusion, few studies have investigated the contribution of generational factors to predicting second-generation health outcomes. Adding grandmaternal risk factors and maternal birth characteristics modestly improved the prediction of SGA and LGA in nulliparous women as compared to models based on maternal clinical factors only. A novel finding of this study is that maternal birthweight z-score may be a useful predictor of abnormal fetal growth. However, predictive performance remained less than optimal, and more research is needed to identify predictors that are readily available early in pregnancy. Future research could build on this work by integrating maternal intergenerational data with grandpaternal and paternal information and other early-pregnancy predictors, including biomarkers and fetal biometric or ultrasound imaging data, which may offer added value when incorporated into an ensemble approach that uses machine learning algorithms.

## Supplementary Information

Supplementary Information.

## Data Availability

The data that support the findings of this study are available from the Reproductive Care Program of Nova Scotia, but restrictions apply to the availability of these data, which were used under license for the current study and are not publicly available. Further queries can be directed to the corresponding author.

## Declarations

### Disclaimer

The Nova Scotia Atlee Perinatal Database (NSAPD) data used in this report were made available by the Reproductive Care Program of Nova Scotia, a program of IWK Health (IWK). Although this analysis is based on data obtained from IWK, and it was reviewed before publication to ensure compliance with NSAPD disclosure policies, and understanding of the variable definitions, the observations and opinions expressed are those of the authors and do not necessarily represent those of IWK.
