# Supplementary material for: Development and validation of super learner models to predict small and large for gestational age in the second generation
Source: Sci Rep. 2025 Sep 26;15:33212. doi: 10.1038/s41598-025-18466-0 (PMC12475498; doi:10.1038/s41598-025-18466-0)
Supplement: Supplementary file 1 — Supplementary Information. [file 41598_2025_18466_MOESM1_ESM.pdf]

Supplementary Information for "Development and validation of Super Learner models to predict small and large for gestational age in the second generation"

AUTHORS: *Mary M. Brown,[1,3] Stefan Kuhle,[2,3] Bruce Smith,[4] Victoria M. Allen,[5] Jennifer Payne,[6] Christy G. Woolcott[3]

AFFILIATIONS: [1]School of Integrated Health, University of New Brunswick, Saint John, NB, Canada; [2]Institute of Clinical Epidemiology, Public Health, Health Economics, Medical Statistics and Informatics, Medical University of Innsbruck, Austria; [3]Perinatal Epidemiology Research Unit, Depts of Obstetrics & Gynaecology and Pediatrics, Dalhousie University, Halifax, NS, Canada; [4]Dept of Mathematics and Statistics, Dalhousie University, Halifax, NS, Canada; [5]Dept of Obstetrics & Gynaecology, Dalhousie University, Halifax, NS, Canada; [6]Dept of Diagnostic Radiology, Dalhousie University, Halifax, NS, Canada

CORRESPONDING AUTHOR: Dr. Mary M. Brown, School of Integrated Health, University of New Brunswick, Saint John, NB, Canada. Email: Maggie.Brown@unb.ca

Supplementary Figure S1. Cross-validated discriminative performance using ROC curves estimated from the Super Learner algorithm for predicting A) small for gestational age and B) large for gestational age fitted using grandmaternal pregnancy-related information and maternal birth characteristics (G0 predictors), maternal clinical factors at 26 weeks' gestation (G1 predictors), and their combination (G0 + G1 predictors).

Supplementary Figure S2. Cross-validated discriminative performance using precision-recall curves estimated from the Super Learner algorithm for predicting A) small for gestational age and B) large for gestational age fitted using grandmaternal pregnancy-related information and maternal birth characteristics (G0 predictors), maternal clinical factors at 26 weeks' gestation (G1 predictors), and their combination (G0 + G1 predictors).

Supplementary Table S1. Details of candidate predictors of infant fetal growth abnormalities.

Supplementary Table S2. Tuning parameter setting, definition, and grid of values assessed for each base learner included in the Super Learner ensemble.

Supplementary Table S3. Cross-validated AUC-PR and AUC-ROC estimates and 95% confidence intervals for the prediction of small for gestational age and large for gestational age.

Supplementary Table S4. Super Learner predicted risk of small for gestational age and large for gestational age fitted using the combined set of predictors (G0 + G1 predictors) and the observed risk estimated from decile groups and pooled across imputed datasets.

Supplementary Table S5. Pooled Super Learner weights across validation folds and corresponding standard errors from prediction models small for gestational age and large for gestational age fitted using the combined set of predictors (G0 + G1 predictors).

Supplementary Table S6. Variable importance ranking for the prediction of small for gestational age and large for gestational age using the top two prediction algorithms from the Super Learner ensemble fitted using the combined set of predictors (G0 + G1 predictors).

Supplementary Table S7. Cross-validated AUC-PR and AUC-ROC estimates and 95% confidence intervals for the prediction of small for gestational age (<3rd percentile for gestational age) and large for gestational age (>97th percentile for gestational age).

Supplementary Table S8. Variable importance ranking for the prediction of small for gestational age (<3rd percentile for gestational age) and large for gestational age (>97th percentile for gestational age) using the top two prediction algorithms from the Super Learner ensemble fitted using the combined set of predictors (G0 + G1 predictors).

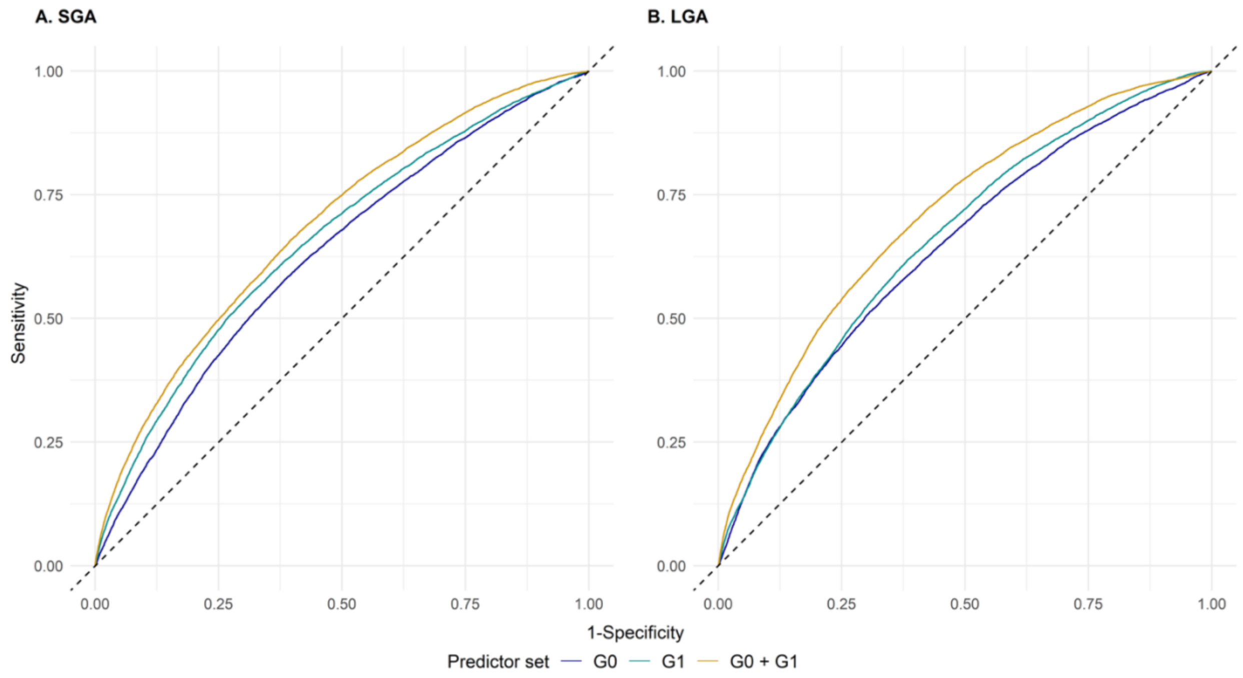

Abbreviations: *LGA* large for gestational age; *ROC* receiver operating characteristic; *SGA* small for gestational age

Supplementary Figure S1. Cross-validated discriminative performance using ROC curves estimated from the Super Learner algorithm for predicting A) small for gestational age and B) large for gestational age fitted using grandmaternal pregnancy-related information and maternal birth characteristics (G0 predictors), maternal clinical factors at 26 weeks' gestation (G1 predictors), and their combination (G0 + G1 predictors). No discrimination is indicated by the dotted line.

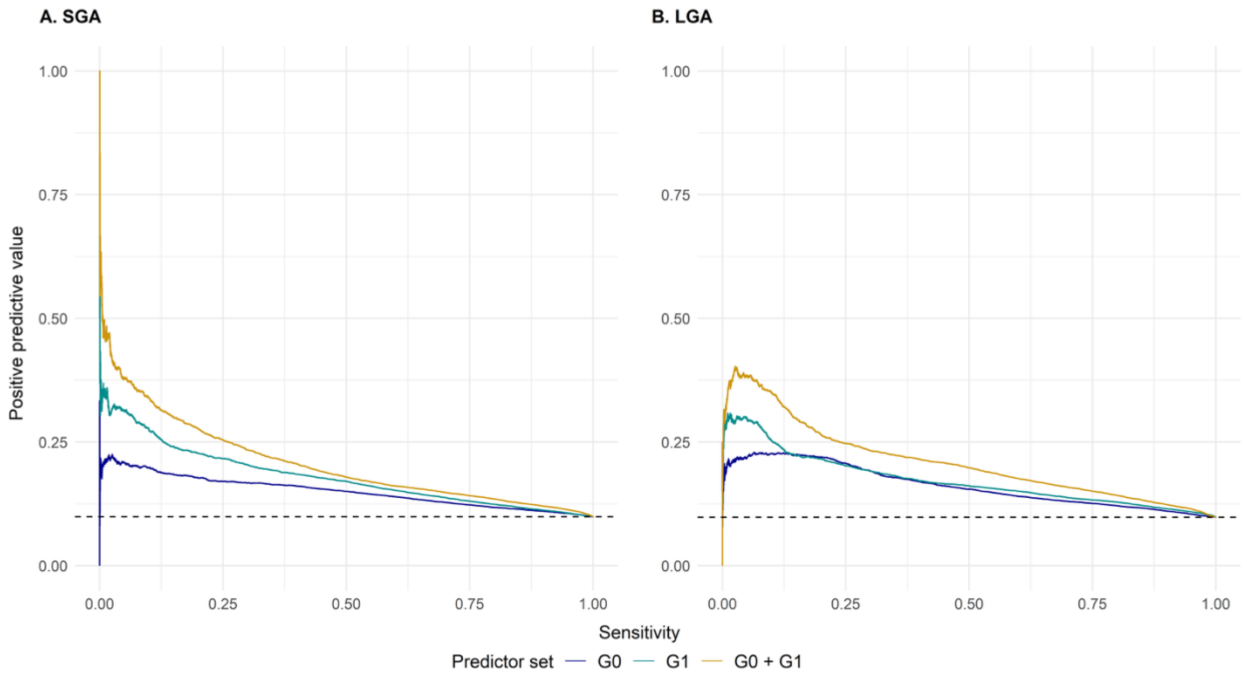

Abbreviations: *AUC-PR* area under the precision-recall curve; *LGA* large for gestational age; *SGA* small for gestational age

Supplementary Figure S2. Cross-validated discriminative performance using precision-recall curves estimated from the Super Learner algorithm for predicting A) small for gestational age and B) large for gestational age fitted using grandmaternal pregnancy-related information and maternal birth characteristics (G0 predictors), maternal clinical factors at 26 weeks' gestation (G1 predictors), and their combination (G0 + G1 predictors). The AUC-PR value that indicates no discrimination is the average prevalence of SGA (9.9%) and LGA (9.9%) in training samples and is indicated by the dotted line.

Supplementary Table S1. Details of candidate predictors of infant fetal growth abnormalities.

Predictors	Type (units or levels)	G0 predictors	G1 predictors
**Sociodemographics**			
Maternal age	Continuous (years)	X	X
Marital status	Binary (married/common-law, other)	X	X
Area-level income quintile	Categorical	X	X
Area of residence	Binary (rural, urban)	X	X
**Pregnancy risk factors**			
Pre-pregnancy body mass index	Continuous (kg/m^2)	X	X
Pre-existing hypertension	Binary (yes, no)	X	X
Pre-existing diabetes	Binary (yes, no)		X
**Pregnancy characteristics**			
Weight gain in pregnancy	Continuous (kg)	X	X
Any smoking in pregnancy	Binary (yes, no)	X	X
Any alcohol use in pregnancy	Binary (yes, no)	X	X
Gestational diabetes	Binary (yes, no)	X	X
Hypertensive disorders of pregnancy	Binary (yes, no)	X	X
Mode of delivery	Binary (vaginal, Caesarean-section)	X	
**Neonatal characteristics**			
Mother's birthweight z-score	Continuous (SD units)	X	
Mother's gestational age at birth	Continuous (weeks)	X	
Infant sex	Binary (male, female)		X

Abbreviations: *SD* standard deviation

Supplementary Table S2. Tuning parameter setting, definition, and grid of values assessed for each base learner included in the Super Learner ensemble.

**Algorithm**	**R package**	**Hyperparameter**		
		Setting	Definition	Values assessed
Generalized additive models	gam	deg.gam	Degrees of freedom	{2, 3, 4}
Elastic net	glmnet	alpha	Elastic net penalty	{0.05, 0.3, 0.7, 0.95}
		lambda	Regularization parameter	Default (100 values)
Random forest[a]	ranger	mtry	Number of covariates (p) sampled for each split	{floor($\sqrt{p}$/2), floor($\sqrt{p}$), p}
		nodesize	Minimum node size	{5, 100, 455}
Extreme gradient boosting	XGBoost	ntrees	Number of trees	{250, 1000}
		max_depth	Maximum depth of a tree	{2, 4}
		shrinkage	Step size shrinkage	{0.05, 2}
Support vector machine	kernlab	C	Regularization parameter	{0.1, 1, 10, 100}

[a] Number of trees constructed for each forest (hyperparameter setting "ntree") set to 2000 for all grid configurations

Supplementary Table S3. Cross-validated AUC-PR and AUC-ROC estimates and 95% confidence intervals for the prediction of small for gestational age and large for gestational age.

Learner	Small for gestational age			Large for gestational age		
	G0 predictors	G1 predictors	G0 + G1 predictors	G0 predictors	G1 predictors	G0 + G1 predictors
**AUC-PR**						
Marginal mean	0.099 (0.099, 0.099)	0.099 (0.099, 0.099)	0.099 (0.099, 0.099)	0.098 (0.098, 0.098)	0.098 (0.098, 0.098)	0.098 (0.098, 0.098)
Logistic regression (main)	0.153 (0.141, 0.166)	0.180 (0.163, 0.199)	0.206 (0.186, 0.228)	0.165 (0.151, 0.180)	0.173 (0.158, 0.190)	0.207 (0.185, 0.230)
Logistic regression (interaction)	0.136 (0.123, 0.152)	0.163 (0.145, 0.183)	0.150 (0.137, 0.164)	0.147 (0.134, 0.162)	0.157 (0.146, 0.170)	0.143 (0.117, 0.172)
SVM	0.110 (0.099, 0.122)	0.113 (0.098, 0.131)	0.147 (0.128, 0.168)	0.116 (0.105, 0.128)	0.127 (0.112, 0.145)	0.147 (0.131, 0.164)
RF	0.147 (0.133, 0.162)	0.170 (0.152, 0.190)	0.190 (0.169, 0.214)	0.162 (0.149, 0.177)	0.173 (0.155, 0.193)	0.206 (0.182, 0.233)
Elastic net	0.154 (0.142, 0.165)	0.181 (0.164, 0.199)	0.208 (0.188, 0.229)	0.166 (0.152, 0.181)	0.175 (0.159, 0.192)	0.211 (0.188, 0.236)
GAM	0.153 (0.141, 0.166)	0.184 (0.166, 0.203)	0.208 (0.187, 0.229)	0.165 (0.150, 0.180)	0.178 (0.160, 0.198)	0.212 (0.190, 0.237)
XGBoost	0.151 (0.137, 0.165)	0.179 (0.157, 0.203)	0.207 (0.187, 0.229)	0.163 (0.149, 0.178)	0.169 (0.148, 0.191)	0.207 (0.184, 0.232)
Discrete Super Learner	0.151 (0.140, 0.164)	0.183 (0.165, 0.202)	0.203 (0.183, 0.225)	0.164 (0.150, 0.179)	0.176 (0.159, 0.195)	0.210 (0.187, 0.236)
Super Learner	0.154 (0.141, 0.168)	0.184 (0.164, 0.206)	0.212 (0.190, 0.234)	0.166 (0.152, 0.180)	0.178 (0.159, 0.198)	0.215 (0.191, 0.241)
**AUC-ROC**						
Marginal mean	0.500 (0.466, 0.534)	0.500 (0.466, 0.534)	0.500 (0.466, 0.534)	0.500 (0.466, 0.534)	0.500 (0.466, 0.534)	0.500 (0.466, 0.534)
Logistic regression (main)	0.625 (0.606, 0.644)	0.654 (0.633, 0.674)	0.681 (0.661, 0.702)	0.644 (0.624, 0.664)	0.661 (0.641, 0.681)	0.699 (0.680, 0.718)
Logistic regression (interaction)	0.595 (0.571, 0.618)	0.630 (0.602, 0.657)	0.619 (0.593, 0.644)	0.617 (0.594, 0.64)	0.636 (0.615, 0.656)	0.597 (0.552, 0.641)
SVM	0.525 (0.495, 0.556)	0.521 (0.483, 0.559)	0.593 (0.565, 0.621)	0.533 (0.51, 0.556)	0.543 (0.514, 0.572)	0.599 (0.572, 0.625)
RF	0.620 (0.599, 0.641)	0.642 (0.621, 0.662)	0.667 (0.646, 0.687)	0.639 (0.617, 0.661)	0.653 (0.633, 0.673)	0.693 (0.673, 0.713)
Elastic net	0.629 (0.609, 0.648)	0.654 (0.633, 0.675)	0.684 (0.663, 0.704)	0.644 (0.624, 0.665)	0.665 (0.645, 0.685)	0.704 (0.685, 0.723)
GAM	0.626 (0.606, 0.645)	0.657 (0.636, 0.678)	0.683 (0.663, 0.703)	0.644 (0.623, 0.663)	0.663 (0.643, 0.683)	0.702 (0.682, 0.721)
XGBoost	0.623 (0.603, 0.643)	0.649 (0.625, 0.672)	0.681 (0.659, 0.701)	0.636 (0.611, 0.660)	0.652 (0.631, 0.673)	0.698 (0.678, 0.718)
Discrete Super Learner	0.627 (0.607, 0.646)	0.656 (0.634, 0.677)	0.679 (0.659, 0.699)	0.641 (0.618, 0.663)	0.659 (0.638, 0.680)	0.701 (0.681, 0.720)
Super Learner	0.628 (0.609, 0.648)	0.657 (0.636, 0.678)	0.686 (0.666, 0.706)	0.644 (0.623, 0.665)	0.662 (0.642, 0.682)	0.706 (0.686, 0.726)

Abbreviations: *AUC-PR* area under the precision-recall curve; *AUC-ROC* area under the receiver operating characteristics curve; *GAM* generalized additive model; *RF* random forest; *SVM* support vector machine; *XGBoost* extreme gradient boosting.

Supplementary Table S4. Super Learner predicted risk of small for gestational age and large for gestational age fitted using the combined set of predictors (G0 + G1 predictors) and the observed risk estimated from decile groups and pooled across imputed datasets.

Decile	Small for gestational age			Large for gestational age		
	n	Predicted risk (%)	Observed risk (%, 95% CI)	n	Predicted risk (%)	Observed risk (%, 95% CI)
1	909.7	2.9	2.2 (0.2, 4.3)	909.8	2.9	2.2 (0.3, 4.2)
2	909.7	4.4	4.3 (2.1, 6.5)	909.7	4.2	3.1 (0.9, 5.2)
3	909.7	5.5	5.9 (3.6, 8.1)	909.6	5.3	5.1 (3.0, 7.2)
4	909.7	6.6	6.7 (4.0, 9.4)	909.7	6.3	6.0 (3.8, 8.2)
5	909.8	7.7	7.8 (5.2, 10.4)	909.8	7.5	7.1 (4.6, 9.6)
6	909.7	9.0	9.4 (6.6, 12.3)	909.6	8.8	8.9 (6.6, 11.1)
7	909.7	10.5	10.7 (7.9, 13.6)	909.7	10.4	10.4 (7.5, 13.3)
8	909.6	12.6	11.7 (8.5, 14.9)	909.7	12.5	13.1 (10.8, 15.5)
9	909.8	15.9	15.2 (12.7, 17.7)	909.7	15.8	17.3 (14.5, 20.1)
10	909.6	24.2	25.2 (22.9, 27.6)	909.7	24.7	24.7 (22.5, 27.0)

Abbreviations: *CI* confidence interval

Supplementary Table S5. Pooled Super Learner weights across validation folds and corresponding standard errors from prediction models small for gestational age and large for gestational age fitted using the combined set of predictors (G0 + G1 predictors).

**Small for gestational age**			**Large for gestational age**		
Learner	Weight	SE	Learner	Weight	SE
XGBoost	0.410	0.134	GAM	0.433	0.132
Elastic net	0.177	0.194	RF	0.251	0.095
GAM	0.176	0.197	XGBoost	0.214	0.117
Logistic regression (main)	0.171	0.198	Elastic net	0.086	0.131
RF	0.034	0.054	SVM	0.009	0.028
Logistic regression (interaction)	0.021	0.023	Logistic regression (interaction)	0.007	0.011
SVM	0.012	0.030	Marginal mean	0.000	0.000
Marginal mean	0.000	0.000	Logistic regression (main)	0.000	0.000

Abbreviations: *GAM* generalized additive model; *RF* random forest; *SE* standard error; *SVM* support vector machine; *XGBoost* extreme gradient boosting

Supplementary Table S6. Variable importance ranking for the prediction of small for gestational age and large for gestational age using the top two prediction algorithms from the Super Learner ensemble fitted using the combined set of predictors (G0 + G1 predictors).

Rank	Predictor	Mean increase in Brier score	Predictor	Mean increase in Brier score
**Small for gestational age**				
	**Extreme gradient boosting**		**Elastic net**	
1	Maternal birthweight z-score	0.0032	Maternal birthweight z-score	0.0024
2	Maternal weight gain in pregnancy at 26 weeks	0.0019	Maternal weight gain in pregnancy at 26 weeks	0.0015
3	Maternal pre-pregnancy BMI	0.0018	Maternal smoking in pregnancy at 26 weeks	0.0013
4	Maternal smoking in pregnancy at 26 weeks	0.0011	Maternal pre-pregnancy BMI	0.0013
5	Maternal area of residence	0.0003	Maternal hypertensive disorders of pregnancy	0.0005
**Large for gestational age**				
	**Generalized additive model**		**Random forest**	
1	Maternal birthweight z-score	0.0044	Maternal birthweight z-score	0.0044
2	Maternal weight gain in pregnancy at 26 weeks	0.0035	Maternal weight gain in pregnancy at 26 weeks	0.0038
3	Maternal pre-pregnancy BMI	0.0020	Maternal pre-pregnancy BMI	0.0033
4	Maternal smoking in pregnancy at 26 weeks	0.0017	Maternal age	0.0018
5	Maternal pre-existing diabetes	0.0005	Maternal smoking in pregnancy at 26 weeks	0.0014

Abbreviations: *BMI* body mass index

Supplementary Table S7. Cross-validated AUC-PR and AUC-ROC estimates and 95% confidence intervals for the prediction of small for gestational age (<3rd percentile for gestational age) and large for gestational age (>97th percentile for gestational age).

**Learner**	**Small for gestational age (<3rd percentile for gestational age)**			**Large for gestational age (>97th percentile for gestational age)**		
	G0 predictors	G1 predictors	G0 + G1 predictors	G0 predictors	G1 predictors	G0 + G1 predictors
**AUC-PR**[a]						
Marginal mean	0.026 (0.026, 0.026)	0.026 (0.026, 0.026)	0.026 (0.026, 0.026)	0.033 (0.033, 0.033)	0.033 (0.033, 0.033)	0.033 (0.033, 0.033)
Logistic regression (main)	0.046 (0.034, 0.062)	0.055 (0.043, 0.071)	0.060 (0.046, 0.079)	0.065 (0.051, 0.081)	0.074 (0.063, 0.087)	0.090 (0.074, 0.109)
Logistic regression (interaction)	0.037 (0.026, 0.052)	0.042 (0.033, 0.054)	0.035 (0.026, 0.049)	0.051 (0.042, 0.062)	0.060 (0.048, 0.075)	0.057 (0.045, 0.073)
SVM	0.032 (0.020, 0.049)	0.029 (0.024, 0.037)	0.037 (0.026, 0.051)	0.036 (0.027, 0.048)	0.047 (0.035, 0.062)	0.063 (0.046, 0.087)
RF	0.041 (0.030, 0.055)	0.047 (0.035, 0.063)	0.054 (0.041, 0.069)	0.065 (0.051, 0.082)	0.080 (0.056, 0.113)	0.088 (0.069, 0.110)
Elastic net	0.046 (0.034, 0.061)	0.054 (0.042, 0.068)	0.059 (0.046, 0.077)	0.069 (0.056, 0.085)	0.076 (0.064, 0.090)	0.092 (0.076, 0.110)
GAM	0.046 (0.035, 0.060)	0.063 (0.045, 0.088)	0.063 (0.047, 0.086)	0.063 (0.051, 0.079)	0.081 (0.066, 0.099)	0.096 (0.077, 0.119)
XGBoost	0.042 (0.030, 0.057)	0.052 (0.040, 0.069)	0.054 (0.042, 0.068)	0.062 (0.049, 0.079)	0.075 (0.054, 0.104)	0.095 (0.071, 0.127)
Discrete Super Learner	0.044 (0.032, 0.061)	0.060 (0.041, 0.087)	0.058 (0.044, 0.075)	0.069 (0.055, 0.085)	0.077 (0.058, 0.102)	0.087 (0.068, 0.109)
Super Learner	0.043 (0.033, 0.056)	0.059 (0.041, 0.083)	0.060 (0.047, 0.076)	0.066 (0.054, 0.082)	0.081 (0.060, 0.107)	0.098 (0.077, 0.125)
**AUC-ROC**						
Marginal mean	0.500 (0.436, 0.564)	0.500 (0.436, 0.564)	0.500 (0.436, 0.564)	0.500 (0.443, 0.557)	0.500 (0.443, 0.557)	0.500 (0.443, 0.557)
Logistic regression (main)	0.614 (0.573, 0.653)	0.658 (0.620, 0.695)	0.680 (0.640, 0.717)	0.641 (0.608, 0.674)	0.693 (0.661, 0.723)	0.733 (0.701, 0.763)
Logistic regression (interaction)	0.544 (0.494, 0.594)	0.598 (0.551, 0.643)	0.547 (0.484, 0.610)	0.592 (0.552, 0.630)	0.636 (0.596, 0.675)	0.601 (0.55, 0.649)
SVM	0.516 (0.449, 0.583)	0.497 (0.429, 0.565)	0.560 (0.476, 0.641)	0.500 (0.435, 0.566)	0.560 (0.512, 0.607)	0.625 (0.577, 0.671)
RF	0.601 (0.555, 0.645)	0.634 (0.596, 0.670)	0.663 (0.625, 0.699)	0.626 (0.583, 0.666)	0.704 (0.671, 0.735)	0.735 (0.705, 0.764)
Elastic net	0.617 (0.578, 0.655)	0.654 (0.616, 0.691)	0.679 (0.639, 0.717)	0.656 (0.623, 0.689)	0.701 (0.669, 0.730)	0.742 (0.711, 0.771)
GAM	0.614 (0.571, 0.655)	0.661 (0.620, 0.699)	0.685 (0.644, 0.723)	0.640 (0.605, 0.673)	0.703 (0.671, 0.733)	0.739 (0.708, 0.768)
XGBoost	0.600 (0.553, 0.645)	0.635 (0.588, 0.679)	0.661 (0.619, 0.701)	0.634 (0.594, 0.672)	0.696 (0.663, 0.727)	0.738 (0.708, 0.766)
Discrete Super Learner	0.613 (0.572, 0.653)	0.657 (0.616, 0.697)	0.674 (0.625, 0.719)	0.656 (0.622, 0.688)	0.702 (0.666, 0.736)	0.734 (0.700, 0.766)
Super Learner	0.610 (0.571, 0.648)	0.657 (0.617, 0.696)	0.683 (0.644, 0.721)	0.652 (0.618, 0.684)	0.710 (0.677, 0.740)	0.750 (0.721, 0.777)

Abbreviations: *AUC-PR* area under the precision-recall curve; *AUC-ROC* area under the receiver operating characteristics curve; *GAM* generalized additive model; *SVM* support vector machine; *RF* random forest; *XGBoost* extreme gradient boosting;

[a] The prevalence of SGA and LGA was 2.6% and 3.3%, respectively, in training samples, which represents the value of no discrimination for AUC-PR.

Supplementary Table S8. Variable importance ranking for the prediction of small for gestational age (<3rd percentile for gestational age) and large for gestational age (>97th percentile for gestational age) using the top two prediction algorithms from the Super Learner ensemble fitted using the combined set of predictors (G0 + G1 predictors).

Rank	Predictor	Mean increase in Brier score	Predictor	Mean increase in Brier score
**Small for gestational age (<3rd percentile for gestational age)**				
	**Generalized additive model**		**Elastic net**	
1	Maternal weight gain in pregnancy at 26 weeks	0.00101	Maternal birthweight z-score	0.00017
2	Maternal pre-pregnancy BMI	0.00050	Maternal smoking in pregnancy at 26 weeks	0.00014
3	Maternal birthweight z-score	0.00037	Maternal weight gain in pregnancy at 26 weeks	0.00009
4	Maternal smoking in pregnancy at 26 weeks	0.00026	Maternal pre-pregnancy BMI	0.00005
5	Maternal gestational hypertension	0.00022	Maternal gestational hypertension	0.00005
**Large for gestational age (>97th percentile for gestational age)**				
	**Random forest**		**Extreme gradient boosting**	
1	Maternal weight gain in pregnancy at 26 weeks	0.00170	Maternal birthweight z-score	0.00087
2	Maternal pre-pregnancy BMI	0.00168	Maternal weight gain in pregnancy at 26 weeks	0.00086
3	Maternal birthweight z-score	0.00122	Maternal pre-pregnancy BMI	0.00057
4	Grandmaternal pre-pregnancy BMI	0.00097	Maternal smoking in pregnancy at 26 weeks	0.00012
5	Grandmaternal age	0.00093	Grandmaternal weight gain in pregnancy at 40 weeks	0.00004

Abbreviations: *BMI* body mass index
